# Predictors of fatigue improvement in multimodal, multimodal-aerobic and aerobic exercise intervention studies in breast cancer survivors with cancer-related fatigue

**DOI:** 10.1038/s41598-025-06701-7

**Published:** 2025-07-01

**Authors:** M. Kröz, M. Reif, L. R. Fässler-Teal, B. Berger, C. Sasselli, R. Zerm, D. Martin, C. Gutenbrunner, A. Büssing

**Affiliations:** 1 Research Institute Havelhöhe, Berlin, Germany; 2 Institute of Integrative Medicine, University of Witten/Herdecke, Witten/Herdecke, Germany; 3 Research Department and Sleep Medicine, Klinik Arlesheim, Arlesheim, Switzerland; 4 Society for Clinical Research, Berlin, Germany; 5 Department of Internal Medicine Havelhöhe Hospital, Berlin, Germany; 6 University Children’s Hospital, Tübingen University, Tübingen, Germany; 7 Clinic for Rehabilitative Medicine, Hannover Medical School, Hannover, Germany

**Keywords:** Autonomic regulation, Breast cancer, Cancer-related fatigue, Internal coherence scale, Multimodal therapy, Salutogenesis, Breast cancer, Quality of life

## Abstract

Cancer-related fatigue (CRF) is common among breast cancer (BC) survivors. In addition to aerobic training, psychoeducation, sleep education/restriction, and mindfulness-based therapies are shown to reduce CRF. This study investigates the predictive effect of hygiogenetic and salutogenetic concepts, such as autonomic regulation (aR), self-regulation (SRS) and internal coherence (ICS) along with sleep quality (PSQI) and quality of life (EORTC QLQ C30, including cognitive, emotional and physical functioning) on the success of CRF therapies. Two studies are analyzed: a pilot (CRF-1) with 36 BC patients and a follow-up study (CRF2) with 126 patients either randomized or assigned to therapy by preference. All parameters were assessed at baseline and 10 weeks post-intervention (T1), and in CRF-2 also six months later (T2), and after four years (T3). Multiple linear regression models were applied. Trait aR and ICS are shown to be significant predictors of CRF when all timepoints of the CRF-2 study are included (β_Trait aR_ = −0.170, df = 70, *p* < 0.001; β_ICS_ = −0.210, df = 70, *p* < 0.01) as well as when combined with data of the CRF-1 study (β_Trait aR_ = −0.144, df = 101, *p* = 0.001; β_ICS_ = −0.211, df = 101, *p* < 0.01). Cognitive Function showed a borderline significance only at T3 and when all CRF-2 study time measurements were combined (β_CF_ = −0.073, df = 70, *p* < 0.05). Using data from two studies with multimodal, aerobic and combined CRF treatments, this study highlights Trait aR and ICS at baseline as long-term predictors of CRF even four years after intervention. A stable autonomic regulation including rest/activity regulation and internal coherence are predictors for therapy response of a multimodal, combination or aerobic treatment in breast cancer survivors with CRF.

**Supplementary Information:**

The online version contains supplementary material available at 10.1038/s41598-025-06701-7.

## Introduction

Cancer-related fatigue (CRF) is a common and distressing symptom complex in cancer patients, found in over 75% of those with metastatic disease^1^ and between 58 and 94% of breast cancer patients undergoing chemotherapy^2^. Both tumor burden and cancer treatment, including chemotherapy^3,4^, radiation therapy^5^, surgery^6^ and anti-hormonal treatments, can impact multiple systems linked to fatigue^7,8^. The discussed pathophysiological alterations are complex, with factors such as serotonin dysregulation, alterations in cellular metabolism, neuroendocrine dysfunction, and inflammation proposed as underlying biological contributors^7,8^, which can be differentiated into central and peripheral hypotheses^9^. Central pathophysiological hypotheses of dysregulation include, in particular, increased cytokines, circadian disorders, disorders of the hypothalamic-pituitary-adrenal axis and vagal afferent or serotonin dysfunction. Peripheral mechanisms include changes in muscle contraction properties^9^. CRF is associated with complex dysregulation, including the loss of circadian rhythm and rest/activity regulation^10^. In turn, this leads to successive dysfunctions and symptoms such as sleep disturbances, fatigue, cognitive dysfunction, reduced memory and concentration, distress, endocrine and autonomic dysregulation^8,11,12^.

Even three to five years after the initial diagnosis and completion of adjuvant treatments have ended CRF still persists in 30–35% of breast cancer survivors^13,14^ and is one of the most prevalent symptoms for cancer survivors who have no evidence of active disease^13,14^. Risk factors for developing CRF can be categorized into predisposing factors, precipitating factors and perpetuating factors^8^. A key research question remains to determine why cancer-related fatigue syndrome becomes chronic and continues to affect about one-third of breast cancer patients for years, even after resolving precipitating (triggering) factors such as standard adjuvant therapies (chemotherapy, radiotherapy, surgery, anti-hormonal treatment) or reducing tumor burden^8^.

The following disorders have previously been discussed as perpetuating factors for CRF: sleep disorders, psychological disorders (e.g. depressive history, trait anxiety, childhood adversities and physical inactivity) and neuroendocrine, autonomic and immunological dysregulation^8^. In integrative medicine, essential health resources include physiological and psychological adaptations that support overall well-being: Physiological adaptation of autonomic functions is a process that promotes *regulation* of bodily systems, as addressed in Hildebrandt’s research on *hygiogenesis*^15,16^. This regulatory process supports the body in maintaining physiological adaptability and resilience and is grounded in principles such as rhythm and e.g. functional normalization. Additionally, successful psychological coping mechanisms are essential, helping individuals find meaning, manage challenges, and achieve a sense of coherence, as described in Antonovsky’s *salutogenesis* model^17,18^. This psycho-social coherence can be linked with physiological regulation to describe more holistic health resources.

Besides the possible perpetuating dysregulative factors reviewed in^8^, we are therefore interested in whether health resources such as health-related quality of life, internal coherence (ICS), sleep quality, autonomic regulation (aR) and self-regulation can serve as predictors for the success of fatigue reduction therapies in breast cancer patients. In addition to the autonomic regulation scale^19^, which is capturing physiological adaption in line with hygiogenetic aspects, salutogenetic (psychological) capacities are to be assessed with the help of the internal coherence scale^20^ and self-regulation scale^21,22^. The study aims to determine the predictive capacity of the various scales for fatigue reduction immediately following a ten-week intervention program, as well as to determine if the scales can predict the success of sustained reduction after six months and four years later. A further objective is to identify the most reliable and robust predictors across the available data by combining time points and studies and investigating whether, and if so, to what extent, the associations between the baseline questionnaires and post-therapy CFS-D differ from those between the baseline questionnaires and baseline CFS-D. The focus is to find questionnaires with reproducible results that do not merely track changes in CFS-D status over time but actually serve as independent predictors of changes in CFS-D throughout the course of treatment.

## Methods

Two studies (CRF-1 and CRF-2) of breast cancer patients with chronic cancer-related fatigue syndrome were included in this exploratory analysis. The CRF-1 pilot study^23^, conducted in 2009, included 36 patients who were assigned to a treatment program based on their preferences. Patients completed questionnaires at baseline (T0) and after a 10-week intervention program (T1). In the longer-term CRF-2 study^24^, conducted between 2011 and including a follow-up until 2017, patients filled out questionnaires at baseline (T0), after the 10-week intervention program (T1), 6 months later (T2) and at a four-year follow-up (T3). The CRF-2 study, which used a comprehensive cohort design (combining a randomized treatment allocation with patient preference), initially involved 105 patients with 84 patients at T1, 81 at T2, and 79 patients who responded at the four-year follow-up (T3).

Both studies compared a 10-week intervention program that included either a multimodal therapy program (MT: psychoeducation, sleep education, restriction and stimulus control, eurythmy therapy, and art therapy) or an aerobic training program (AT). Additionally, in the CRF-2 study, a third arm with a combination therapy (CT) of both MT and AT was included.

Both studies were conducted at the Research Institute Havelhöhe (FIH), Berlin and the Hannover Medical School. In the CRF-2 study a third center at the University of Witten/Herdecke was included. Both CRF-1 and CRF-2 studies were investigator-initiated trials which were conducted according to the declaration of Helsinki. Breast cancer patients with chronic cancer-related fatigue syndrome following adjuvant treatment were included. For further details of inclusion and exclusion criteria see^23,24^.

### Ethics approval statement 

Data was available from the CRF-1 and CRF-2 studies, which followed the guidelines for clinical trials (Declaration of Helsinki, ICH-GCP). The CRF-1 study was approved by the ethics committees of the Medizinische Hochschule Hannover (ethics reference number: 5257–2009, 3.3.2009). The CRF2-study was approved by the ethics committee of “Ärztekammer Berlin” (ethics reference number: ETH-06/11, 23.05.2011, with an amendment on 27.04.2015) and confirmed by the ethics committees of the Medizinische Hochschule Hannover (ethics reference number: 1119–2011, 27.06.2011, with an amendment on 23.06.2015) and the University of Witten/Herdecke (ethics reference number: 125/2011, 25.10.2011, with an amendment on 12.11.2015). The study was subjected to GCP-conform monitoring and all included patients signed a written consent. The study is registered in German Clinical Trials Register (DRKS-ID: DRKS00003736; Date of registering 19.06.2012).

### Interventions

Comparable interventions were carried out in both studies.

#### Multimodal therapy (MT)

The multimodal therapy incorporates four components: psychoeducation, sleep education, eurythmy therapy and art therapy.

Psychoeducation included 8 sessions that provided information on breast cancer and cancer-related fatigue, dealing with distressing feelings and thoughts, promoting physical and mental health, the importance of communication and social support, personal responsibility, concentration, stress management exercises and strategies for reorientation to support patient self-management in dealing with CRF^23,24^.

Sleep education consisted of two sessions that provided information about the circadian rhythm, the basic rest/activity rhythm, restful and disturbed sleep, associated changes in daytime functions, and principles of sleep hygiene. Based on two fortnightly sleep diaries that patients filled out at baseline and two weeks later, each patient received information about general breast cancer related sleep changes, as well as individualized recommendations for moderate sleep restriction, and stimulus control^24–27^.

Eurythmy therapy is a mindfulness-oriented movement therapy, which is usually conducted in anthroposophic medicine as part of inpatient and outpatient care. During eurythmy therapy, sounds are expressed in gestures and movements. During both studies a defined series of exercises, such as I-A-O, clenching - spreading or the so-called “cancer series” were used^23,24,28^.

Art therapy sessions began with a short section of form drawing and continued with watercolor painting. Within the studies, a developmental series of painting was included starting with darkness and progressing to daylight over the course of the sessions^23,24,29^.

#### Aerobic training (AT)

The intervention consisting solely of aerobic training was used as the control arm of the studies. Using heart rate monitors exercise was conducted around 70–80% of the pre-determined maximal heart rate^30^. Aerobic training was conducted under the guidance of a trainer and included an additional individualized home-based training program for participants to follow^23,24^.

#### Combination therapy (CT)

The combination therapy (CT) was implemented only in the CRF2 study and integrated all four components of the MT with the aerobic training (AT)^24^.

Further details of the different therapies are described in^23,24^.

### Outcomes

In both CRF-1 and CRF-2 studies psychometric and physiological measures were captured using questionnaires at baseline (T0) and after ten weeks of intervention (T1). In the CRF-2 study questionnaires were re-evaluated six months (T2) and four years (T3) after the end of the intervention^23,24^. The following questionnaires were evaluated:

Cancer Fatigue Scale - German version (CFS-D)

The CFS-D is a 15-item questionnaire that uses a five-point Likert scale^31^ to assess fatigue and is structured into three subscales: “physical fatigue”, “cognitive fatigue” and “affective fatigue”. Originally developed in Japan^32^ the questionnaire has since been validated in several languages^33–35^, including German. The scale is a robust measure with strong internal consistency and good test-retest reliability as well as sufficient validity^31,32^. It is recommended as one of the multidimensional scales for measuring cancer-related fatigue^36^. CFS-D scores range from zero to 60, with a cut-off at 22/23 distinguishing between no/low and moderate fatigue^37^.

#### Trait autonomic regulation (Trait aR)

The Trait aR questionnaire includes 18 items on a 3-point Likert scale. It consists of three subscales “Orthostatic-Circulatory Regulation”, “Rest-Activity Regulation” and “Digestive Regulation” with satisfactory internal consistency and good test-retest reliability^19,38^. High scores indicate a higher aR, with the scale ranging from 18 to 54^19,38^. The Trait aR scale is considered as a psychometric measure with the potential to capture hygiogenetic autonomic function^15,19^.

#### Internal coherence scale (ICS)

The ICS is a compact questionnaire with ten items on a 5-point Likert scale. It is formed on the basis of a factor analysis of two subscales, an eight-item “Inner resilience and coherence” subscale and a two-item “Thermo coherence” subscale. The ICS has robust internal consistency and good test-retest reliability^20^ and is validated up to an age of 96 years^39^. Higher values indicate higher internal coherence, with the scale ranging from 10 to 50^20^.

#### Self-regulation (SRS)

The Self-regulation Scale (SRS) is a short 16-item Self-regulation Scale^21,22^. It measures self-regulation on a 6-point Likert scale via two subscales: (1) Change of Behavior to achieve goal and (2) Achieve Satisfaction and Well-being. Together the sub-scales form the sum score. The SRS is a valid and reliable self-report instrument with robust internal consistency (Cronbach’s α of *r* = 0.95) and satisfying to good retest reliability (rtt = 0.82)^22^.

#### European organization of research and treatment in cancer quality of life questionnaire (EORTC QLQ C30)

The EORCT QLC-C30 is a widely used cancer-specific scale for measuring health-related quality of life (HRQL) multi-dimensionally based on a 30-item core questionnaire. It is a standard instrument consisting of five functional scales, a subscale for global health status and nine symptom scales^40^. The two items that make up the Global Health Status/QoL scale are scored on a 7-point Likert scale, while all other items are scored on a 4-point Likert scale. All subscales are converted to a percentage scale (0–100%) to allow for comparisons. In the functional and global health/QoL scales, higher values indicate better HRQL, whereas in the symptom scales, higher values represent greater symptom burden. Most subscales of the EORTC QLQ C30 demonstrate acceptable to good internal consistency, sufficient validity and a robust classification basis^40–42^. The subscales include measures of Cognitive, Emotional and Physical Functioning.

#### Pittsburgh sleep quality index (PSQI)

The Pittsburgh Sleep Quality Index (PSQI) is a widely used questionnaire that assesses sleep quality based on 19 self-rated items in 7 subscales which together form a total score ranging between 0 and 21^43^. The PSQI shows sufficient reliability and validity^43^.

### Statistics

All data were analysed using linear regression analysis, with CFS-D as the dependant/outcome variable and all questionnaire scores (potential predictors) as continuous explanatory variables. The independent factors ‘treatment’ (AT vs. MT vs. CT), ‘study center’ (Berlin vs. Hannover vs. Herdecke), and ‘study arm allocation’ (random vs. preference; relevant for the CRF-2 study) were included to mirror the general study design.

#### Primary analysis: determination of potential predictors

To establish and compare the predictive effects of the different questionnaires over the course the four years, data from the different time points of measurement (T1 = end of intervention, T2 = 6 months after intervention, T3 = 4 year follow up) were first analyzed in separate models (M1, M2, M3). Subsequently, in order to identify the most reliable and reproducible predictors while accounting for temporal effects, data from all three CRF-2 study measurements (T1, T2, and T3) were included as dependent variables in a single model (M_Total_) and ‘time point’ (T1, T2, T3) was added as a factor. To address the potential autocorrelation from assessing the same patients at different time points in the combined model, a mixed-effects model with a repeated-measures structure was implemented in M_Total_, using a first-order autoregressive covariance pattern. Finally, the data from both studies (CRF-1, CRF-2) were combined into a comprehensive model (M_comb_), with an additional fixed factor ‘study’ to control for the data source (CRF-1 vs. CRF-2).

For all regression models (M_Total_ and M_comb,_ M1-M3), the slopes of each regression parameter and the explained partial variance (η²) were calculated to measure the strength of association between each predictor and the CFS-D scale. All covariates were checked for multi-collinearity by partial correlation analysis, yet only negligible degrees of multi-collinearity were observed.

To enable direct comparison between all slopes and variance components within and between models, all predictor scales were rescaled to a uniform range of 0 to 100. The standardized slope of a given questionnaire thus represents the amount of change in the CFS-D scale corresponding to a one-percent increase in the independent scale.

Simple regression analyses were conducted separately for all models (M_Total_ and M_Comb,_ M1-M3 univariate), and for each predictor and time point as sensitivity assessments. An auxiliary model was used to compare two questionnaires. The auxiliary model included a binary variable called the “predictor index”, which indicated which questionnaire was being analyzed. Predictor values from both questionnaires were incorporated, as well as an interaction term between the “predictor index” and the predictor values. The purpose of the interaction term is to estimate the difference in slopes between the predictors of the two questionnaires^44^.

#### Consolidating analysis: identification of predictors with strongest association with CFS-D by pairwise comparisons

In order to compare the strength of associations between the questionnaire scales and CFS-D, statistical comparisons between predictor scales were conducted by estimating the mean difference and corresponding standard error between the slopes of two predictors and then calculating the p-value (t-test within the linear mixed model) and respective confidence interval.

In the multivariate analyses these differences could be calculated directly from estimated regression slopes of the predictor scales. In the univariate analyses, an auxiliary model was used to compare two questionnaires. The auxiliary model included a binary variable called the “predictor index,” which indicated which questionnaire was being analyzed. Predictor values from both questionnaires were incorporated, as well as an interaction term between the “predictor index” and the predictor values, with the interaction term representing the estimated difference in slopes between the predictors of the two questionnaires^44^.

#### Supportive analysis: comparison of relationships with CFS-D at baseline (T0) and follow-up visits (T1, T2, T3)

In an additional analysis, the association of each potential predictor on CFS-D at the end of treatment (T1) and at the two follow ups (T2, T3) was compared to the relationship with CFS-D at baseline. The aim was to identify questionnaires, which did not simply track changes with CFS-D status over time but actually served as independent predictors for the changes in CFS-D throughout the course of the treatment. To do this, a regression model was applied, analogous to the ones for determining predictor scales (M_Total_ and M_Comb_, M1-M3). The models included a binary factor indicating whether the CFS-D measurement was taken at baseline or follow-up. Changes in slopes between baseline and end of treatment were estimated by the interaction term ‘time x predictor value’^44^. This analysis was conducted using both multiple regression models, which included all potential predictors along with their interactions with time, and simple regression models, which examined only one predictor at a time alongside its interaction term with the time of measurement. In the analyses including all visits of the CRF-2 study, the dependency between measurements of the same patient was again accounted for by a first-order autoregressive covariance pattern.

All tests were performed two-sided at an alpha error level of 5%. Since the analyses were exploratory, no adjustments were made for multiple testing. All analyses were carried out using SAS/STAT software, Version 9.4 of the SAS System for Windows (SAS Institute Inc., Cary, NC, USA, 2016).

## Results

### Primary analysis: determination of potential predictors

The results of the CRF-1 study have been published elsewhere^45^ and are reproduced in the appendix (Table S1) for comparison. In the CRF-2 study, simple regression analysis showed significant relationships between all questionnaires and CFS-D for all models (Table S3) when each questionnaire was considered on its own. Considered in the multivariate analysis, regression coefficients of all questionnaires on CFS-D were relatively small at the first time-point of measurement immediately after the ten-week therapy period (T1, Table S2) and no significant associations with CRF were evident (Table S2). Stronger associations developed at the second measurement point after six months (T2, Table S2) for the questionnaires on autonomic regulation (Trait aR) and the Internal Coherence Scale (ICS). Regression slopes of the multivariate model more than doubled in size from T1 (β_Trait aR_ = -0.116, df = 65, p = n.s.; β_ICS_ = -0.117, df = 65, p = n.s., Table S2) to T2 (β_Trait aR_ = -0.249, df = 61, *p* < 0.001; β_ICS_ = -0.275, df = 61, *p* < 0.05, Table S2). Although the strength of these associations slightly decreased by the measurement at the four-year follow-up (T3), the statistical significance could still be corroborated for both Trait aR and ICS (M3: β_Trait aR_ = -0.159, df = 62, *p* < 0.05; β_ICS_ = -0.232, df = 62, *p* < 0.05; Table S2). None of the other questionnaires showed a significant regression coefficient in any of the three multivariate analyses except for a borderline significance of (*p* = 0.046) in the Cognitive Functioning scale at the 4-year follow-up (T3, Table S2).

In the model including all time-points of the CRF-2 study (M_Total_), Trait aR and ICS once again showed significant slopes with CFS-D (Table 1). Their standardized values were at least three times larger than those of all other covariates (M_Total_: β_Trait aR_ = -0.155, df = 99, *p* < 0.01; β_ICS_ = -0.242, df = 99, *p* < 0.01; Table 1). Cognitive functioning (CF) also reached a significant value when all time measurements were combined in M_Total_ (β_CF_ = -0.073, df = 70, *p* < 0.05; Table 1). The same outcome was observed for Trait aR and ICS when data of the CRF-1 study were included (M_comb_: β_Trait aR_ = -0.144, df = 101, *p* = 0.001; β_ICS_ = -0.211, df = 101, *p* < 0.01; Table 1). However, CF was again non-significant (Table 1).

**Table 1:** Results from the multiple regression analysis of model M_Total_ (CRF-2 all time points combined) and M_Comb_ (CRF-1 and CRF-2 studies combined) showing regression coefficients (Regr Coeff), Standard Errors (SE), adjusted R-squared (adj. *R*^2^), explained partial variance (partial η^2^), standardized regression coefficients (Stand. Reg. Coeff), degrees of freedom (df), t-value (t) and p-values (*p*).

Model	Var.	Regr coeff	SE	adj. *R*^2^	Partial η^2^	Stand. Regr. coeff	Fully stand. regr. coeff β	df	t	*p*
M_Total_	PF	−0.055	0.043	0.39	0.014	−0.055	−0.099	70	−1.29	n.s.
	EF	0.005	0.041		0.000	0.005	0.011	70	0.12	n.s.
	**CF**	**−0.073**	**0.033**		**0.033**	**−0.073**	**−0.211**	**70**	**−2.21**	< 0.05 (*)
	PSQI	0.037	0.041		0.004	0.037	0.071	70	0.89	n.s.
	**Trait aR**	**−0.473**	**0.129**		**0.088**	**−0.170**	**-0.294**	**70**	**−3.67**	< 0.001 (***)
	**ICS**	**−0.524**	**0.186**		**0.053**	**−0.210**	**−0.288**	**70**	**−2.82**	< 0.01 (**)
	SRS	0.020	0.058		0.002	0.020	0.032	70	0.34	n.s.
M_Comb_	PF	−0.064	0.036	0.40	0.018	−0.064	−0.119	101	−1.78	< 0.1 (†)
	EF	0.011	0.035		0.000	0.011	0.026	101	0.32	n.s.
	CF	−0.050	0.027		0.022	−0.050	−0.146	101	−1.85	< 0.1 (†)
	PSQI	0.277	0.172		0.010	0.058	0.112	101	1.61	n.s.
	**Trait aR**	**−0.399**	**0.118**		**0.069**	**−0.144**	**−0.242**	**101**	**−3.38**	= 0.001 (***)
	**ICS**	**−0.527**	**0.165**		**0.054**	**−0.211**	**−0.292**	**101**	**−3.20**	< 0.01 (**)
	SRS	−0.859	1.015		0.001	−0.043	−0.069	101	−0.85	n.s.

Significant values are in bold. The dependent variable is CFS-D, independent variables (Var.) are: *PF*  physical functioning,  *EF * emotional functioning, *CF *  cognitive functioning, *PSQI * Pittsburgh sleep quality index, *Trait aR * Trait autonomic regulation, *ICS * Internal Coherence Scale, *SRS*  self-regulation.

Across all time measurements of the CRF-2 study Trait aR and ICS showed the largest amount of explained partial variance η² in both simple and multiple regression analysis (M_Total_: Trait aR scale: η² = 0.088; ICS scale: η² = 0.053; Table 1). However, the degree of explained variance remains far lower than the largest absolute values observed in the CRF-1 study (multiple regression analysis: SRS scale: η² = 0.349; ICS scale: η² = 0.190; Table S1)^45^.

### Consolidating analysis: identification of predictors with strongest association with CFS-D by pairwise comparisons of strength of associations

The ICS scale showed the strongest association with CFS-D in absolute terms (Table 1, M_Total_ and M_Comb_). In the pairwise comparisons of the bivariate regression models including all data of the CRF-2 study, the strength of the ICS slope differed significantly from the slopes of the EORTC QLQ-C30 Emotional, Cognitive and Physical Functioning scales as well as from the slope of the PSQI total score (Table S4a). However, in the pairwise comparisons of the multiple regression model the ICS slope showed a significant difference only to the slope of the Emotional Functioning scale (Table 2a). Whereas in the same model the Trait aR scale showed a significantly steeper slope compared with the Emotional Functioning and the SRS scales, likely due to the smaller standard errors of the estimated differences (Table 2a). Including the data from the CRF-1 study into the analysis of slope differences distinctly increased the number of significant differences, but again this was primarily for the Trait aR and ICS scales (Tables 2b, S4b). In the bivariate models additional significant differences were found for ICS vs. SRS, and for Trait aR vs. Cognitive Functioning, Emotional Functioning and PSQI (Table S4b); in the multivariate model for ICS vs. Cognitive Functioning, SRS and Physical Functioning, and for Trait aR vs. Cognitive Functioning, Physical Functioning and PSQI; Table 2).

**Table 2:**
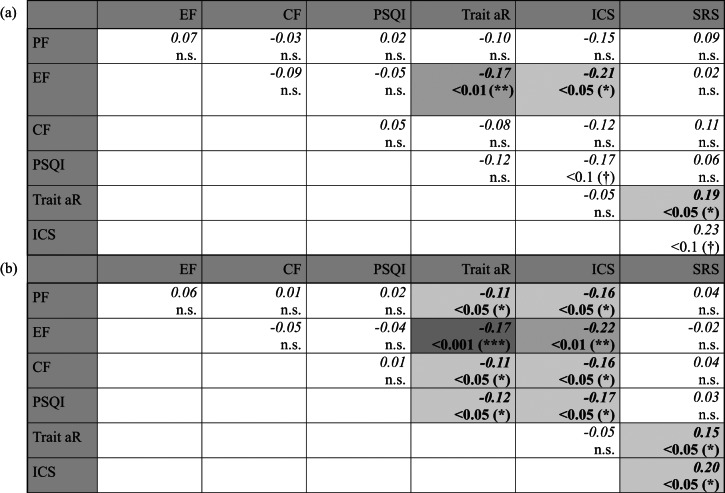
Pairwise comparisons of standardized regression coefficients on CFS-D between questionnaires (*PF*  physical functioning,  *EF * emotional functioning, *CF * cognitive functioning, *PSQI*  pittsburgh sleep quality index, *Trait aR * trait autonomic regulation, *ICS*  internal coherence Scale, *SRS* = self-regulation scale), showing absolute differences in slope (upper value) and p-values (lower value), respectively. All significant differences are highlighted in bold and shaded according to significance (light grey: *p* < 0.05, medium grey: *p* < 0.01, dark grey: *p* < 0.001). Results from (a) CRF-2 study all time-points, multivariate model, (b) all studies combined, multivariate model.

### Supportive analysis: comparison of relationships with CRF at baseline (T0) and follow-up visits (T1, T2, T3)

The full results of the supportive analysis are presented in the supplementary material (Table S5a-f). In the CRF-1 study multiple regression analyses showed that only the SRS and ICS scales had a significant regression relationship with CFS-D following the therapy intervention (T1), while no such association was found with baseline CFS-D (Table S5a^45^), . In contrast, in the CRF-2 study this pattern was observed for the Trait aR and ICS scales across T2 (Table S5c) and T3 (Table S5d), with SRS showing no significance at any time-point (Tables S5b-d). Using the full data of the CRF2 study combined (Table S2, M_Total_), the regression slope of the Trait aR scale tripled in size from baseline to follow-up, indicating a statistically significant increase in the relationship (Trait aR multiple regression coefficients at baseline (BL) and from short to long term (T1-3): β_BL_ = -0.060, β_end_ = -0.177, change (± standard error) in coefficients: Δ_end−baseline_ = -0.117 ± 0.053, df = 250, *p* < 0.05; Table S2). The ICS scale, on the other hand, showed a significant relationship with the CFS-D already at baseline and subsequently at T2 and T3 measurements (Table S5c and S5d), whereas the relationship between SRS and CFS-D scales was negligible over all follow-up visits (Table S5b-d). The association between ICS with CFS-D remained stable across all time points (Baseline, T1, T2, T3; Table 3), indicating the robust predictive capacity of this scale; whereas, the relationship between baseline Trait aR and CFS-D measured at baseline and post-baseline increased significantly. In contrast, the other questionnaires showed a descriptive weakening of their association with CFS-D over time (Table 3). This pattern remained stable even when the data of the CRF-1 study were included in the analysis (Table 3). These findings highlight the particular relevance of ICS and Trait aR as potentially reliable predictive markers for long-term changes in CRF. The slightly discrepant results of the CRF-1 study when considered on its own is likely a result of the larger sample size of the CRF-2 study, which is due to both the higher number of patients and the repeated questionnaire assessments.

**Table 3:** Results from the multivariate regression analysis on differences in regression coefficients of different independent variables (Var.) on CFS-D at baseline (BL-CRF) and follow-up (End-CRF) visits, showing standardized regression coefficients (Stand. Regr Coeff) with baseline (BL) and end CFS-D and corresponding standard errors (SE) and p-values (*p*), as well as standardised regression coefficients of the differences with corresponding SE, degrees of freedom (df), t-value (t) and p-values (*p*). The dependent variable is CFS-D, independent variables (Var.) are: *PF *  physical functioning, *EF *= emotional functioning, *CF*  cognitive functioning, *PSQI * pittsburgh sleep quality index, *Trait aR*  trait autonomic regulation, *ICS *  internal coherence scale, *SRS*  self-regulation scale. Results from (M_Total_) CRF-2 study (T1-T3) and (M_Comb_) CRF-2 study (T1-T3) and CRF-1 study combined.

	Var.	Stand. Regr-Coeff BL-CRF	SE	*p*-value (symbol)	Stand. Regr-Coeff End-CRF	SE	*p*-value (symbol)	Stand Regr-Coeff difference	SE	df	t-value	*p*-value BL vs. End
M_Total_	PF	−0.108	0.034	< 0.01 (**)	−0.046	0.034	n.s.	0.062	0.047	248	1.31	n.s.
	EF	−0.017	0.031	n.s.	−0.001	0.034	n.s.	0.016	0.046	270	0.36	n.s.
	CF	−0.155	0.025	< 0.001 (***)	−0.080	0.026	< 0.01 (**)	0.075	0.036	261	2.09	< 0.05 (*)
	PSQI	0.047	0.033	n.s.	−0.022	0.033	n.s.	-0.069	0.046	257	−1.50	n.s.
	Trait aR	−0.060	0.038	n.s.	−0.177	0.038	< 0.001 (***)	−0.117	0.053	250	−2.20	< 0.05 (*)
	ICS	−0.200	0.062	< 0.01 (**)	−0.196	0.058	< 0.001 (***)	0.004	0.083	234	0.04	n.s.
	SRS	0.086	0.048	< 0.1 (†)	0.017	0.046	n.s.	-0.070	0.065	245	−1.08	n.s.
M_Comb_	PF	−0.080	0.027	< 0.01 (**)	−0.045	0.031	n.s.	0.034	0.041	349	0.84	n.s.
	EF	−0.019	0.025	n.s.	0.001	0.030	n.s.	0.021	0.039	362	0.53	n.s.
	CF	−0.134	0.019	< 0.001 (***)	−0.058	0.023	< 0.05 (*)	0.076	0.030	360	2.56	< 0.05 (*)
	PSQI	0.010	0.027	n.s.	−0.032	0.031	n.s.	−0.042	0.041	353	−1.05	n.s.
	Trait aR	−0.049	0.033	n.s.	−0.170	0.036	< 0.001 (***)	−0.121	0.048	345	−2.51	< 0.05 (*)
	ICS	−0.184	0.051	< 0.001 (***)	−0.201	0.054	< 0.001 (***)	−0.017	0.073	337	−0.24	n.s.
	SRS	0.033	0.039	n.s.	−0.034	0.043	n.s.	−0.066	0.057	344	−1.17	n.s.

## Discussion

Trait autonomic regulation and internal coherence were found to be the only two consistently significant predictors of prospective medium and longer-term fatigue development in breast cancer patients with chronic CRF taking into account different treatment approaches (multimodal, aerobic and combination therapy). Although no predictors could be identified for fatigue development immediately post intervention in the CRF-2 study, both Trait aR and ICS showed potential to be able to predict cancer fatigue development over the longer term at 6 months and even 4 years after intervention. Cognitive functioning was shown to have only a borderline significant relationship to CFS-D with the relationship stronger at the four-year follow-up than earlier and is therefore not considered to be a likely key predictor. Adding data from the CRF-1 study, which were only short-term data (T1) and did not include the combination therapy approach, appeared to weaken the relationship of CF with CFS-D.

The results raise a number of questions about the role of health resource-related factors, such as autonomic regulation (hygiogenetic)^19^ and internal coherence (salutogenetic)^20,39^. The interventions studied - ranging from endurance training to adjustments to sleep schedules, stress and self-management and mindfulness - combine hygiogenetic and salutogenetic approaches that have previously been shown to lead to sustained improvement in CRF^46^. The present analysis indicates that in breast cancer survivors with CRF, trait autonomic regulation, a hygiogenetic marker, can be identified as a predictor of fatigue improvement in the presence of chronic CRF. This can be assessed using the Trait aR questionnaire and by evaluating associated functions such as rest/activity regulation, orthostatic-circulatory regulation (including acral blood flow), circadian well-being and digestive regulation, focusing on both stability and adaptability^19^. The evening decline in core body temperature, accompanied by an increase in distal temperature, is associated with increased sleepiness and faster sleep onset^47^. This supports the findings of an observational study, in which good autonomic regulation predicts low global fatigue and cognitive fatigue after six years^48^. Better autonomic regulation is therefore associated with improved fatigue outcomes in breast cancer patients. Psychometric and to some extent physiological rest/activity regulation can be improved through interventions^49^. Disturbed sleep^50,51^, rhythm disturbances^7,52–54^ reduced rest/activity amplitude and phase delays (trend towards eveningness)^54,55^ are commonly observed in patients with clinically relevant CRF and are often accompanied by increased autonomic dysregulation^56^.

Cold feet are related with a prolongation of sleep-onset latency of 15 min^57^. Patients with vasospasticity show prolonged sleep-onset latency and more nocturnal disturbances after sleep initiation^58^. In breast cancer patients, feeling cold, indicative of deficient thermoregulation, and having cold extremities is associated with complex dysregulations^59^. Acral coldness has also been reported in premorbid phases of life^60^. Acral coldness is associated with orthostatic intolerance, cardiovascular issues and autonomic dysregulation^61^. Clinically, both reduced orthostatic regulation – indicating orthostatic intolerance^62^ – and acral coldness are linked with CRF in breast cancer patients^63^ a pattern similarly observed in chronic fatigue patients with post-COVID syndrome^61^. In summary, the literature confirms that low autonomic regulation may be a vulnerability factor for long-term cancer-related fatigue, whereas high autonomic regulation appears predictive of reduced fatigue and a favorable treatment response. Therapies, such as the multimodal approach, that strengthen patients’ autonomic regulation and, in particular their rest/activity regulation^49^, may therefore show higher effectiveness in reducing chronic cancer fatigue^46^.

Inner resilience and coherence with items such as ‘to be able to face the day with confidence’, ‘confident enough to solve problems’, ‘coming up with good solutions’, ‘the doing and inner wishes was consistent’, ‘deep down I felt secure’ is captured by a high level of internal coherence and is associated with thermo-coherence^20^. Besides autonomic regulation, a hygiogenetic marker, the present study also found internal coherence, a salutogenetic marker, to be a significant factor influencing long-term fatigue. There are a couple of studies published on the conceptually related Sense of Coherence Scale (SOC) and its impact in terms of mortality, health, morbidity and symptom burden in patients with cancer^64–66^. A high sense of coherence was associated with a 31% reduction in overall mortality and a 27% reduction in cancer mortality after up to 6 years^64^. A low sense of coherence is also prospectively associated with a 52% higher cancer incidence after eight years, while the effect is reduced to 14% after 12 years^65^. This was particularly true for people over 55 years of age (65%)^65^. Wainwright et al. discussed that lifestyle choices can be a potential pathway linking SOC and general health^66^. A high sense of coherence may also have a delaying effect on the occurrence of cancer^65^. In an exercise trial, breast cancer patients with a higher SOC had a better perceived health including less fatigue, less symptom burden and a better health-related quality of life (HRQL)^67^. Rohani et al. also found that in breast cancer patients a higher SOC was a mediator process for HRQL, cognitive and social functions as well as a partial mediator for fatigue^68^. As with autonomic regulation, the literature therefore supports the link between sense of coherence or internal coherence and chronic cancer fatigue and suggests that boosting internal coherence will be beneficial for treatment response and reducing chronic fatigue in cancer patients.

Intervention studies have already shown that internal coherence can be improved. Cancer fatigue patients showed higher internal coherence following ten weeks of intervention therapies^69^, whereby multimodal and combination therapies showed a higher sustained success of fatigue reduction over aerobic training on its own. In an observational clinical registry of 231 breast cancer patients treated initially with conventional adjuvant therapy and a multimodal integrative medicine approach, 12-month follow-up data showed associations towards improvements in ‘internal coherence’, ‘inner resilience and coherence’, and ‘thermo coherence’ after nursing compresses. ‘Internal coherence’, ‘inner resilience and coherence’ also showed associations towards improvements after music therapy and ‘elaborate consultation and life review’ and finally ‘inner resilience and coherence’ improved following rhythmic massage^70^. Given the predictive capacity of the ICS and the effectiveness of therapies in improving ICS^69^, there is strong motivation to incorporate ICS-targeted therapies, such as multimodal and combination therapies^69^ in treating cancer-related fatigue.

Our study supports existing literature, indicating that both hygiogenetic (Trait aR) and salutogenic (ICS) health resources can act as positive influencing factors on predisposing and perpetuating factors in chronic fatigue syndrome (CRF). Incorporating these health resources into treatment programs is likely to significantly improve treatment outcomes. In comparison to previous observational studies, this study offers reproducible, robust, and sustainable results through a series of main and sensitivity regression analyses that align with health-resource-based working principles. Variability between the different interventions was minimized by standardizing therapy delivery following a detailed manual. Patient adherence to the therapies was tracked using diaries. Detailed descriptions of therapy delivery, as well as measures of adherence during and after the intervention, are provided and discussed in^46^. Furthermore, interventions across various partner institutions were highly standardized, following a detailed manual with a defined session schedule, which likely contributes to the consistency of our findings. On the other hand, there are limitations to consider, including the relatively low number of patients and the inclusion of only two studies in the analyses^46^. Additionally, all reported studies come from a single research group and focus only on breast cancer patients with chronic CRF and without metastases, which may limit the generalizability of the findings to other cancer types or other chronic conditions. Finally, the existence of an underlying, hitherto undetected confounder which may have influenced aR, ICS and fatigue scales on separate causal pathways cannot completely be ruled out.

## Conclusion

In conclusion, this article demonstrates that autonomic regulation and internal coherence might be independent predictors of improvement in chronic, long-term cancer-related fatigue. Future studies should further investigate the impact of these hygiogenetic and salutogenetic psychometric measures to deepen our understanding of the underlying working-principles of non-pharmacological treatments, such as mindfulness-oriented therapies, multimodal interventions and integrative medicine based pharmacological approaches for managing cancer-related fatigue and other chronic conditions involving complex dysregulations. Such studies could allow better indications of how these scales and predictors can be applied in routine care to tailor interventions and treatment approaches.

## Electronic supplementary material

Below is the link to the electronic supplementary material.

Supplementary Material 1

## Data Availability

Any request for data should be made in writing to the corresponding author, and these will be considered.
