# Correction: Predictors of fatigue improvement in multimodal, multimodal-aerobic and aerobic exercise intervention studies in breast cancer survivors with cancer-related fatigue

**DOI:** 10.1038/s41598-025-14140-7

**Published:** 2025-08-05

**Authors:** M. Kröz, M. Reif, L. R. Fässler-Teal, B. Berger, C. Sasselli, R. Zerm, D. Martin, C. Gutenbrunner, A. Büssing

**Affiliations:** 1 Research Institute Havelhöhe, Berlin, Germany; 2 Institute of Integrative Medicine, University of Witten/Herdecke, Witten/Herdecke, Germany; 3 Research Department and Sleep Medicine, Klinik Arlesheim, Arlesheim, Switzerland; 4 Society for Clinical Research, Berlin, Germany; 5 Department of Internal Medicine Havelhöhe Hospital, Berlin, Germany; 6 University Children’s Hospital, Tübingen University, Tübingen, Germany; 7 Clinic for Rehabilitative Medicine, Hannover Medical School, Hannover, Germany

Correction to: *Scientific Reports* 10.1038/s41598-025-06701-7, published online 1 July 2025

The Ethics approval statement in the original version of this Article was omitted. The Ethics approval statement now reads:

 “Data was available from the CRF-1 and CRF-2 studies, which followed the guidelines for clinical trials (Declaration of Helsinki, ICH-GCP). The CRF-1 study was approved by the ethics committees of the Medizinische Hochschule Hannover (ethics reference number: 5257-2009, 3.3.2009). The CRF2-study was approved by the ethics committee of “Ärztekammer Berlin” (ethics reference number: ETH-06/11, 23.05.2011, with an amendment on 27.04.2015) and confirmed by the ethics committees of the Medizinische Hochschule Hannover (ethics reference number: 1119-2011, 27.06.2011, with an amendment on 23.06.2015) and the University of Witten/Herdecke (ethics reference number: 125/2011, 25.10.2011, with an amendment on 12.11.2015). The study was subjected to GCP-conform monitoring and all included patients signed a written consent. The study is registered in German Clinical Trials Register (DRKS-ID: DRKS00003736; Date of registering 19.06.2012).”

Additionally, the Acknowledgments section was incomplete. The Acknowledgments section now reads:

“The authors thank all CRF study group collaborators for their contributions. The CRF-1 and CRF-2 studies were funded by the following foundations: Mahle Stifung, Stuttgart, Germany, Christophorus Stifung, Stuttgart, Germany, Dr. Hauschka Stifung, Bad Boll/Eckwälden, Germany, Stiftung Helixor, Rosenfeld, Germany, Gyllenberg Foundation, Helsinki, Finland. The foundations were not involved in the study design, the collection, analysis or interpretation of the data. MK and RZ received financial support by Humanus Institute, Berlin, Germany and Software AG Stiftung, Darmstadt, Germany.”

The original Article has been corrected.

