# Supplementary material for: Predictors of fatigue improvement in multimodal, multimodal-aerobic and aerobic exercise intervention studies in breast cancer survivors with cancer-related fatigue
Source: Sci Rep. 2025 Jul 1;15:20690. doi: 10.1038/s41598-025-06701-7 (PMC12218493; doi:10.1038/s41598-025-06701-7)
Supplement: Supplementary file 1 — Supplementary Material 1 [file 41598_2025_6701_MOESM1_ESM.docx]

**Supplementary Information**

Table S1: Results from the multiple regression (Mv) and univariate (Uv) regression analysis of the CRF-1 study showing Regression Coefficients (Regr Coeff), Standard Errors (SE), adjusted R-squared (adj. R^2^), explained partial variance (partial η2), standardized Regression Coefficients (Stand. Reg. Coeff), degrees of freedom (df), t-value (t) and p-values (p). The dependent variable is CFS-D, independent variables (Var.) are: PF = physical functioning, EF = emotional functioning, CF = cognitive functioning, PSQI = Pittsburgh sleep quality index, Trait aR = Trait autonomic regulation, ICS = Internal Coherence Scale, SRS = self-regulation [46]

| **Model** | **Var.** | **Regr Coeff** | **SE** | **adj. R^2^** | **partial** η**^2^** | **Stand. Regr. Coeff** | **Fully Stand. Regr. Coeff β** | **df** | **t** | **p** |
| --- | --- | --- | --- | --- | --- | --- | --- | --- | --- | --- |
| **Mv** | PF | -0.081 | 0.066 | 0.66 | 0.067 | -0.081 | -0.180 | 21 | -1.23 | 0.2319 |
|  | EF | 0.068 | 0.074 | . | 0.039 | 0.068 | 0.174 | 21 | 0.92 | 0.3669 |
|  | CF | -0.046 | 0.043 | . | 0.050 | -0.046 | -0.142 | 21 | -1.05 | 0.3054 |
|  | PSQI | 0.591 | 0.303 | . | 0.153 | 0.124 | 0.238 | 21 | 1.95 | 0.0649 |
|  | aR | 0.153 | 0.319 | . | 0.011 | 0.055 | 0.076 | 21 | 0.48 | 0.6371 |
|  | **ICS** | -0.672 | 0.303 | . | 0.190 | -0.269 | -0.397 | 21 | -2.22 | **0.0376** |
|  | **SRS** | -6.373 | 1.900 | . | 0.349 | -0.319 | -0.517 | 21 | -3.35 | **0.0030** |
| **Uv** | **PF** | -0.198 | 0.074 | 0.18 | 0.211 | -0.198 | -0.441 | 27 | -2.69 | **0.0122** |
| **Uv** | **EF** | -0.150 | 0.069 | 0.12 | 0.149 | -0.150 | -0.383 | 27 | -2.17 | **0.0387** |
| **Uv** | **CF** | -0.123 | 0.054 | 0.13 | 0.163 | -0.123 | -0.385 | 27 | -2.29 | **0.0301** |
| **Uv** | PSQI | 0.638 | 0.452 | 0.03 | 0.069 | 0.134 | 0.257 | 27 | 1.41 | 0.1693 |
| **Uv** | **aR** | -0.835 | 0.334 | 0.16 | 0.188 | -0.301 | -0.417 | 27 | -2.50 | **0.0190** |
| **Uv** | **ICS** | -1.270 | 0.221 | 0.53 | 0.551 | -0.508 | -0.751 | 27 | -5.76 | **<.0001** |
| **Uv** | **SRS** | -8.494 | 1.753 | 0.45 | 0.465 | -0.425 | -0.689 | 27 | -4.84 | **<.0001** |

Table S2: Results from the multiple regression analysis of model M1 (CRF-2 study at T1), M2 (CRF-2 study at T2) and M3 (CRF-2 study at T3) showing Regression Coefficients (Regr Coeff), Standard Errors (SE), adjusted R-squared (adj. R^2^), explained partial variance (partial η^2^), standardized Regression Coefficients (Stand. Reg. Coeff), degrees of freedom (df), t-value (t) and p-values (p). The dependent variable is CFS-D, independent variables (Var.) are: PF = physical functioning, EF = emotional functioning, CF = cognitive functioning, PSQI = Pittsburgh sleep quality index, Trait aR = Trait autonomic regulation, ICS = Internal Coherence Scale, SRS = self-regulation

| **Model** | **Var.** | **Regr Coeff** | **SE** | **adj. R^2^** | **partial** η**^2^** | **Stand. Regr. Coeff** | **Fully Stand. Regr. Coeff β** | **df** | **t** | **p** |
| --- | --- | --- | --- | --- | --- | --- | --- | --- | --- | --- |
| **M1** | PF | -0.089 | 0.061 | 0.31 | 0.031 | -0.089 | -0.175 | 65 | -1.45 | 0.1512 |
|  | EF | 0.008 | 0.058 | . | 0.000 | 0.008 | 0.020 | 65 | 0.14 | 0.8925 |
|  | CF | -0.068 | 0.047 | . | 0.031 | -0.068 | -0.216 | 65 | -1.45 | 0.1528 |
|  | PSQI | 0.364 | 0.278 | . | 0.026 | 0.076 | 0.161 | 65 | 1.31 | 0.1954 |
|  | Trait aR | -0.323 | 0.182 | . | 0.046 | -0.116 | -0.220 | 65 | -1.78 | 0.0801 |
|  | ICS | -0.293 | 0.258 | . | 0.020 | -0.117 | -0.176 | 65 | -1.14 | 0.2597 |
|  | SRS | -0.311 | 1.602 | . | 0.001 | -0.016 | -0.028 | 65 | -0.19 | 0.8467 |
| **M2** | PF | -0.052 | 0.064 | 0.46 | 0.010 | -0.052 | -0.089 | 61 | -0.80 | 0.4269 |
|  | EF | -0.057 | 0.062 | . | 0.014 | -0.057 | -0.130 | 61 | -0.92 | 0.3615 |
|  | CF | -0.041 | 0.048 | . | 0.012 | -0.041 | -0.115 | 61 | -0.85 | 0.3961 |
|  | PSQI | 0.011 | 0.294 | . | 0.000 | 0.002 | 0.004 | 61 | 0.04 | 0.9707 |
|  | **Trait aR** | **-0.691** | **0.193** | **.** | **0.173** | **-0.249** | **-0.413** | **61** | **-3.57** | **0.0007** |
|  | **ICS** | **-0.688** | **0.271** | **.** | **0.095** | **-0.275** | **-0.362** | **61** | **-2.54** | **0.0138** |
|  | SRS | 0.628 | 1.694 | . | 0.002 | 0.031 | 0.049 | 61 | 0.37 | 0.7121 |
| **M3** | PF | -0.068 | 0.066 | 0.35 | 0.017 | -0.068 | -0.114 | 62 | -1.03 | 0.3052 |
|  | EF | 0.022 | 0.064 | . | 0.002 | 0.022 | 0.044 | 62 | 0.34 | 0.7346 |
|  | **CF** | **-0.102** | **0.050** | **.** | **0.063** | **-0.102** | **-0.270** | **62** | **-2.04** | **0.0460** |
|  | PSQI | 0.049 | 0.304 | . | 0.000 | 0.010 | 0.019 | 62 | 0.16 | 0.8722 |
|  | **Trait aR** | **-0.441** | **0.195** | **.** | **0.076** | **-0.159** | **-0.262** | **62** | **-2.27** | **0.0270** |
|  | **ICS** | **-0.579** | **0.286** | **.** | **0.062** | **-0.232** | **-0.303** | **62** | **-2.03** | **0.0470** |
|  | SRS | 1.943 | 1.813 | . | 0.018 | 0.097 | 0.149 | 62 | 1.07 | 0.2881 |

Table S3: Results from the univariate regression analysis of models M1-M3, M_Total_ and M_Comb_ showing Regression Coefficients (Regr Coeff), Standard Errors (SE), adjusted R-squared (adj. R^2^), explained partial variance (partial η2), standardized Regression Coefficients (Stand. Reg. Coeff), degrees of freedom (df), t-value (t) and p-values (p). The dependent variable is CFS-D, independent variables (Var.) are: PF = physical functioning, EF = emotional functioning, CF = cognitive functioning, PSQI = Pittsburgh sleep quality index, Trait aR = Trait autonomic regulation, ICS = Internal Coherence Scale, SRS = self-regulation.

| **Model** | **Var.** | **Regr Coeff** | **SE** | **adj. R^2^** | **partial** η**^2^** | **Stand. Regr. Coeff** | **Fully Stand. Regr. Coeff β** | **df** | **t** | **p** |
| --- | --- | --- | --- | --- | --- | --- | --- | --- | --- | --- |
| **M1 univariate** | PF | -0.206 | 0.057 | 0.14 | 0.148 | -0.206 | -0.407 | 75 | -3.61 | 0.0005 |
|  | EF | -0.143 | 0.050 | 0.09 | 0.099 | -0.143 | -0.371 | 75 | -2.87 | 0.0053 |
|  | CF | -0.168 | 0.035 | 0.23 | 0.237 | -0.168 | -0.536 | 75 | -4.83 | <.0001 |
|  | PSQI | 0.850 | 0.255 | 0.12 | 0.131 | 0.178 | 0.376 | 74 | 3.34 | 0.0013 |
|  | aR | -0.693 | 0.165 | 0.17 | 0.192 | -0.249 | -0.472 | 74 | -4.19 | <.0001 |
|  | ICS | -0.753 | 0.175 | 0.19 | 0.199 | -0.301 | -0.453 | 75 | -4.31 | <.0001 |
|  | SRS | -3.667 | 1.219 | 0.09 | 0.110 | -0.183 | -0.325 | 73 | -3.01 | 0.0036 |
| **M2 univariate** | PF | -0.227 | 0.071 | 0.12 | 0.128 | -0.227 | -0.394 | 70 | -3.20 | 0.0020 |
|  | EF | -0.227 | 0.055 | 0.18 | 0.196 | -0.227 | -0.515 | 70 | -4.14 | <.0001 |
|  | CF | -0.199 | 0.040 | 0.25 | 0.263 | -0.199 | -0.556 | 70 | -4.99 | <.0001 |
|  | PSQI | 0.924 | 0.311 | 0.09 | 0.114 | 0.194 | 0.358 | 69 | 2.97 | 0.0041 |
|  | aR | -1.009 | 0.186 | 0.28 | 0.298 | -0.363 | -0.602 | 69 | -5.41 | <.0001 |
|  | ICS | -1.122 | 0.184 | 0.34 | 0.347 | -0.449 | -0.591 | 70 | -6.10 | <.0001 |
|  | SRS | -5.525 | 1.380 | 0.17 | 0.189 | -0.276 | -0.429 | 69 | -4.01 | 0.0002 |
| **M3 univarate** | PF | -0.203 | 0.066 | 0.11 | 0.121 | -0.203 | -0.342 | 70 | -3.10 | 0.0028 |
|  | EF | -0.164 | 0.052 | 0.11 | 0.123 | -0.164 | -0.328 | 70 | -3.14 | 0.0025 |
|  | CF | -0.202 | 0.038 | 0.28 | 0.289 | -0.202 | -0.537 | 70 | -5.33 | <.0001 |
|  | PSQI | 0.664 | 0.300 | 0.05 | 0.065 | 0.139 | 0.263 | 70 | 2.21 | 0.0300 |
|  | aR | -0.756 | 0.189 | 0.17 | 0.189 | -0.272 | -0.449 | 69 | -4.00 | 0.0002 |
|  | ICS | -0.836 | 0.188 | 0.21 | 0.220 | -0.334 | -0.438 | 70 | -4.45 | <.0001 |
|  | SRS | -3.420 | 1.476 | 0.07 | 0.072 | -0.171 | -0.262 | 69 | -2.32 | 0.0235 |
| **M_Total_ univariate** | PF | -0.200 | 0.049 | 0.12 | 0.126 | -0.200 | -0.359 | 80 | -4.11 | <.0001 |
|  | EF | -0.164 | 0.039 | 0.12 | 0.132 | -0.164 | -0.378 | 80 | -4.23 | <.0001 |
|  | CF | -0.188 | 0.028 | 0.25 | 0.257 | -0.188 | -0.539 | 80 | -6.80 | <.0001 |
|  | PSQI | 0.827 | 0.219 | 0.09 | 0.098 | 0.174 | 0.336 | 79 | 3.77 | 0.0003 |
|  | aR | -0.820 | 0.133 | 0.22 | 0.220 | -0.295 | -0.510 | 79 | -6.19 | <.0001 |
|  | ICS | -0.920 | 0.135 | 0.25 | 0.250 | -0.368 | -0.505 | 80 | -6.80 | <.0001 |
|  | SR | -4.339 | 1.039 | 0.12 | 0.119 | -0.217 | -0.350 | 78 | -4.18 | <.0001 |
| **M_Comb_ univariate** | PF | -0.196 | 0.040 | 0.13 | 0.135 | -0.196 | -0.362 | 111 | -4.85 | <.0001 |
|  | EF | -0.157 | 0.034 | 0.12 | 0.135 | -0.157 | -0.365 | 111 | -4.59 | <.0001 |
|  | CF | -0.170 | 0.025 | 0.23 | 0.240 | -0.170 | -0.494 | 111 | -6.81 | <.0001 |
|  | PSQI | 0.750 | 0.199 | 0.08 | 0.094 | 0.157 | 0.304 | 110 | 3.77 | 0.0003 |
|  | aR | -0.768 | 0.127 | 0.19 | 0.213 | -0.276 | -0.467 | 110 | -6.03 | <.0001 |
|  | ICS | -0.953 | 0.120 | 0.28 | 0.280 | -0.381 | -0.528 | 111 | -7.92 | <.0001 |
|  | SRS | -4.762 | 0.933 | 0.14 | 0.146 | -0.238 | -0.384 | 109 | -5.11 | <.0001 |

Table S4: Pairwise comparisons of differences in standardized regression coefficients on CFS-D between questionnaires (PF = physical functioning, EF = emotional functioning, CF = cognitive functioning, PSQI = Pittsburgh sleep quality index, Trait aR = Trait autonomic regulation, ICS = Internal Coherence Scale, SRS = self-regulation scale), showing: Diff = absolute differences in slope and p-values respectively. All significant differences are highlighted in bold and shaded according to significance (light grey: p < 0.05, medium grey: p < 0.01, dark grey: p < 0.001). Results from (a) CRF2-study all timepoints, univariate models, (b) all studies combined, univariate models,

|  | EF | CF | PSQI | Trait aR | ICS | SRS |
| --- | --- | --- | --- | --- | --- | --- |
| PF | *0.03*  *0.6759* | *-0.00*  *0.9764* | *0.03*  *0.6829* | *-0.10*  *0.1622* | ***-0.17***  ***0.0301*** | *-0.01*  *0.8769* |
| EF |  | *-0.03*  *0.6164* | *0.00*  *0.9830* | *-0.13*  *0.0560* | ***-0.19***  ***0.0067*** | *-0.04*  *0.5537* |
| CF |  |  | *0.03*  *0.6000* | *-0.10*  *0.0949* | ***-0.17***  ***0.0119*** | *-0.02*  *0.8026* |
| PSQI |  |  |  | *-0.13*  *0.0685* | ***-0.20***  ***0.0065*** | *-0.04*  *0.5823* |
| Trait aR |  |  |  |  | *-0.07*  *0.3826* | *0.09*  *0.2442* |
| ICS |  |  |  |  |  | *0.16*  *0.0514* |

(a)

(b)

|  | EF | CF | PSQI | Trait aR | ICS | SRS |
| --- | --- | --- | --- | --- | --- | --- |
| PF | *0.01*  *0.8045* | *0.01*  *0.8338* | *0.02*  *0.6901* | *-0.12*  *0.0572* | ***-0.22***  ***0.0009*** | *-0.08*  *0.2243* |
| EF |  | *-0.00*  *0.9729* | *0.01*  *0.8470* | ***-0.13***  ***0.0237*** | ***-0.23***  ***0.0002*** | *-0.09*  *0.1215* |
| CF |  |  | *0.01*  *0.7778* | ***-0.13***  ***0.0163*** | ***-0.23***  ***<0.0001*** | *-0.09*  *0.0947* |
| PSQI |  |  |  | ***-0.14***  ***0.0243*** | ***-0.24***  ***0.0003*** | *-0.10*  *0.1492* |
| Trait aR |  |  |  |  | *-0.10*  *0.1492* | *0.04*  *0.5272* |
| ICS |  |  |  |  |  | ***0.14***  ***0.0407*** |

Table S5: Results from the uni- (Uv) and multivariate (Mv) regression analysis on differences in regression coefficients of different independent variables (Var.) on CFS-D at baseline (BL-CRF) and follow-up (End-CRF) visits, showing Standardized Regression Coefficients (Stand. Regr Coeff) with baseline (BL) and end CFS-D and corresponding Standard Errors (SE) and p-values (p), as well as Standardised Regression Coefficients of the differences with corresponding SE, degrees of freedom (df), t-value (t) and p-values (p). The dependent variable is CFS-D, independent variables (Var.) are: PF = physical functioning, EF = emotional functioning, CF = cognitive functioning, PSQI = Pittsburgh sleep quality index, Trait aR = Trait autonomic regulation, ICS = Internal Coherence Scale, SRS = self-regulation scale. Results from (a) CRF-1 study (T1), (b) CRF-2 study (T1), (c) CRF-2 study (T2), (d) CRF-2 study (T3), (e) univariate results of M_Total_ (above double line) and M_Comb_ (below double line).

(a)

|  | **Var.** | **Stand. Regr-Coeff BL-CRF** | **SE** | **p-value (symbol)** | **Stand. Regr-Coeff End-CRF** | **SE** | **p-value (symbol)** | **Stand Regr-Coeff Difference** | **SE** | **df** | **t-value** | **p-value BL vs End** |
| --- | --- | --- | --- | --- | --- | --- | --- | --- | --- | --- | --- | --- |
| **Mv** | PF | -0.077 | 0.027 | <0.01 (**) | -0.116 | 0.066 | <0.1 (†) | -0.039 | 0.071 | 30.9 | -0.55 | 0.5847 |
|  | EF | -0.021 | 0.025 | n.s. | 0.027 | 0.074 | n.s. | 0.048 | 0.078 | 27.7 | 0.61 | 0.5452 |
|  | CF | -0.136 | 0.019 | <0.001 (***) | -0.044 | 0.045 | n.s. | 0.093 | 0.049 | 30.9 | 1.89 | 0.0680 |
|  | PSQI | 0.018 | 0.027 | n.s. | -0.117 | 0.066 | <0.1 (†) | -0.135 | 0.071 | 29.7 | -1.90 | 0.0676 |
|  | aR | -0.050 | 0.033 | n.s. | 0.106 | 0.116 | n.s. | 0.156 | 0.121 | 26.1 | 1.29 | 0.2079 |
|  | ICS | -0.181 | 0.052 | <0.001 (***) | -0.236 | 0.123 | <0.1 (†) | -0.055 | 0.133 | 30.6 | -0.42 | 0.6806 |
|  | SRS | 0.024 | 0.040 | n.s. | -0.310 | 0.099 | <0.01 (**) | -0.334 | 0.106 | 29.6 | -3.14 | 0.0038 |
| **Uv** | PF | -0.220 | 0.033 | <0.001 (***) | -0.208 | 0.072 | <0.01 (**) | 0.012 | 0.080 | 42.2 | 0.16 | 0.8762 |
| **Uv** | EF | -0.169 | 0.027 | <0.001 (***) | -0.159 | 0.069 | <0.05 (*) | 0.010 | 0.074 | 37.6 | 0.14 | 0.8922 |
| **Uv** | CF | -0.211 | 0.017 | <0.001 (***) | -0.123 | 0.053 | <0.05 (*) | 0.088 | 0.056 | 35.1 | 1.58 | 0.1233 |
| **Uv** | PSQI | -0.086 | 0.039 | <0.05 (*) | -0.130 | 0.095 | n.s. | -0.044 | 0.103 | 38.6 | -0.43 | 0.6687 |
| **Uv** | aR | -0.222 | 0.043 | <0.001 (***) | -0.284 | 0.122 | <0.05 (*) | -0.062 | 0.129 | 35.4 | -0.48 | 0.6356 |
| **Uv** | ICS | -0.379 | 0.040 | <0.001 (***) | -0.505 | 0.089 | <0.001 (***) | -0.126 | 0.097 | 40.2 | -1.30 | 0.2010 |
| **Uv** | SRS | -0.202 | 0.043 | <0.001 (***) | -0.428 | 0.091 | <0.001 (***) | -0.226 | 0.101 | 39.9 | -2.25 | 0.0303 |

(b)

|  | **Var.** | **Stand. Regr-Coeff BL-CRF** | **SE** | **p-value (symbol)** | **Stand. Regr-Coeff End-CRF** | **SE** | **p-value (symbol)** | **Stand Regr-Coeff Difference** | **SE** | **df** | **t-value** | **p-value BL vs End** |
| --- | --- | --- | --- | --- | --- | --- | --- | --- | --- | --- | --- | --- |
| **Mv** | PF | -0.078 | 0.027 | <0.01 (**) | -0.043 | 0.057 | n.s. | 0.035 | 0.063 | 103 | 0.56 | 0.5763 |
|  | EF | -0.023 | 0.025 | n.s. | 0.024 | 0.057 | n.s. | 0.047 | 0.063 | 95.8 | 0.76 | 0.4507 |
|  | CF | -0.136 | 0.019 | <0.001 (***) | -0.077 | 0.045 | <0.1 (†) | 0.058 | 0.049 | 94.7 | 1.19 | 0.2355 |
|  | PSQI | 0.017 | 0.027 | n.s. | -0.058 | 0.057 | n.s. | -0.075 | 0.063 | 100 | -1.19 | 0.2368 |
|  | aR | -0.051 | 0.033 | n.s. | -0.131 | 0.065 | <0.05 (*) | -0.080 | 0.072 | 105 | -1.10 | 0.2733 |
|  | ICS | -0.174 | 0.052 | <0.01 (**) | -0.130 | 0.097 | n.s. | 0.044 | 0.108 | 108 | 0.41 | 0.6816 |
|  | SRS | 0.024 | 0.039 | n.s. | -0.034 | 0.077 | n.s. | -0.058 | 0.086 | 104 | -0.68 | 0.4986 |
| **Uv** | PF | -0.220 | 0.033 | <0.001 (***) | -0.166 | 0.055 | <0.01 (**) | 0.055 | 0.063 | 139 | 0.86 | 0.3901 |
| **Uv** | EF | -0.169 | 0.027 | <0.001 (***) | -0.129 | 0.049 | <0.01 (**) | 0.040 | 0.055 | 128 | 0.73 | 0.4697 |
| **Uv** | CF | -0.211 | 0.017 | <0.001 (***) | -0.164 | 0.034 | <0.001 (***) | 0.047 | 0.038 | 117 | 1.22 | 0.2238 |
| **Uv** | PSQI | -0.089 | 0.039 | <0.05 (*) | -0.166 | 0.052 | <0.01 (**) | -0.077 | 0.064 | 159 | -1.21 | 0.2297 |
| **Uv** | aR | -0.218 | 0.043 | <0.001 (***) | -0.252 | 0.059 | <0.001 (***) | -0.034 | 0.072 | 154 | -0.47 | 0.6361 |
| **Uv** | ICS | -0.374 | 0.040 | <0.001 (***) | -0.300 | 0.067 | <0.001 (***) | 0.075 | 0.078 | 134 | 0.96 | 0.3390 |
| **Uv** | SRS | -0.202 | 0.042 | <0.001 (***) | -0.193 | 0.059 | <0.01 (**) | 0.009 | 0.072 | 151 | 0.12 | 0.9044 |

(c)

|  | **Var.** | **Stand. Regr-Coeff BL-CRF** | **SE** | **p-value (symbol)** | **Stand. Regr-Coeff End-CRF** | **SE** | **p-value (symbol)** | **Stand Regr-Coeff Difference** | **SE** | **df** | **t-value** | **p-value BL vs End** |
| --- | --- | --- | --- | --- | --- | --- | --- | --- | --- | --- | --- | --- |
| **Mv** | PF | -0.078 | 0.027 | <0.01 (**) | 0.005 | 0.061 | n.s. | 0.083 | 0.066 | 93 | 1.25 | 0.2130 |
|  | EF | -0.021 | 0.025 | n.s. | -0.043 | 0.062 | n.s. | -0.022 | 0.067 | 86.2 | -0.33 | 0.7402 |
|  | CF | -0.135 | 0.019 | <0.001 (***) | -0.063 | 0.047 | n.s. | 0.071 | 0.051 | 86.8 | 1.41 | 0.1633 |
|  | PSQI | 0.014 | 0.027 | n.s. | 0.030 | 0.061 | n.s. | 0.016 | 0.067 | 90 | 0.24 | 0.8128 |
|  | aR | -0.050 | 0.033 | n.s. | -0.258 | 0.070 | <0.001 (***) | -0.208 | 0.078 | 93.1 | -2.67 | 0.0089 |
|  | ICS | -0.181 | 0.052 | <0.001 (***) | -0.251 | 0.103 | <0.05 (*) | -0.070 | 0.114 | 96.7 | -0.62 | 0.5372 |
|  | SRS | 0.028 | 0.039 | n.s. | -0.017 | 0.083 | n.s. | -0.045 | 0.090 | 93.8 | -0.50 | 0.6190 |
| **Uv** | PF | -0.221 | 0.033 | <0.001 (***) | -0.170 | 0.068 | <0.05 (*) | 0.051 | 0.075 | 112 | 0.68 | 0.4972 |
| **Uv** | EF | -0.167 | 0.027 | <0.001 (***) | -0.215 | 0.054 | <0.001 (***) | -0.049 | 0.061 | 111 | -0.80 | 0.4254 |
| **Uv** | CF | -0.210 | 0.017 | <0.001 (***) | -0.201 | 0.039 | <0.001 (***) | 0.009 | 0.043 | 102 | 0.21 | 0.8351 |
| **Uv** | PSQI | -0.093 | 0.039 | <0.05 (*) | -0.161 | 0.064 | <0.05 (*) | -0.068 | 0.074 | 126 | -0.92 | 0.3611 |
| **Uv** | aR | -0.219 | 0.043 | <0.001 (***) | -0.356 | 0.067 | <0.001 (***) | -0.137 | 0.079 | 131 | -1.73 | 0.0858 |
| **Uv** | ICS | -0.375 | 0.040 | <0.001 (***) | -0.437 | 0.073 | <0.001 (***) | -0.062 | 0.083 | 118 | -0.75 | 0.4548 |
| **Uv** | SRS | -0.199 | 0.042 | <0.001 (***) | -0.292 | 0.067 | <0.001 (***) | -0.093 | 0.078 | 131 | -1.20 | 0.2340 |

(d)

|  | **Var.** | **Stand. Regr-Coeff BL-CRF** | **SE** | **p-value (symbol)** | **Stand. Regr-Coeff End-CRF** | **SE** | **p-value (symbol)** | **Stand Regr-Coeff Difference** | **SE** | **df** | **t-value** | **p-value BL vs End** |
| --- | --- | --- | --- | --- | --- | --- | --- | --- | --- | --- | --- | --- |
| **Mv** | PF | -0.076 | 0.027 | <0.01 (**) | -0.058 | 0.062 | n.s. | 0.018 | 0.068 | 93.4 | 0.27 | 0.7891 |
|  | EF | -0.021 | 0.025 | n.s. | 0.017 | 0.062 | n.s. | 0.038 | 0.067 | 88.9 | 0.58 | 0.5645 |
|  | CF | -0.135 | 0.019 | <0.001 (***) | -0.111 | 0.047 | <0.05 (*) | 0.024 | 0.051 | 89 | 0.47 | 0.6410 |
|  | PSQI | 0.017 | 0.027 | n.s. | -0.006 | 0.060 | n.s. | -0.023 | 0.066 | 93.5 | -0.35 | 0.7264 |
|  | aR | -0.051 | 0.033 | n.s. | -0.157 | 0.067 | <0.05 (*) | -0.106 | 0.075 | 98.8 | -1.42 | 0.1576 |
|  | ICS | -0.181 | 0.052 | <0.001 (***) | -0.200 | 0.105 | <0.1 (†) | -0.020 | 0.115 | 99 | -0.17 | 0.8650 |
|  | SRS | 0.025 | 0.039 | n.s. | 0.071 | 0.084 | n.s. | 0.046 | 0.092 | 96.2 | 0.50 | 0.6190 |
| **Uv** | PF | -0.219 | 0.033 | <0.001 (***) | -0.190 | 0.063 | <0.01 (**) | 0.029 | 0.071 | 117 | 0.40 | 0.6866 |
| **Uv** | EF | -0.167 | 0.027 | <0.001 (***) | -0.165 | 0.051 | <0.01 (**) | 0.003 | 0.057 | 117 | 0.05 | 0.9629 |
| **Uv** | CF | -0.210 | 0.017 | <0.001 (***) | -0.204 | 0.036 | <0.001 (***) | 0.006 | 0.040 | 108 | 0.16 | 0.8730 |
| **Uv** | PSQI | -0.088 | 0.039 | <0.05 (*) | -0.134 | 0.060 | <0.05 (*) | -0.046 | 0.071 | 135 | -0.65 | 0.5177 |
| **Uv** | aR | -0.218 | 0.042 | <0.001 (***) | -0.269 | 0.066 | <0.001 (***) | -0.051 | 0.078 | 133 | -0.65 | 0.5194 |
| **Uv** | ICS | -0.376 | 0.040 | <0.001 (***) | -0.332 | 0.072 | <0.001 (***) | 0.044 | 0.082 | 119 | 0.54 | 0.5932 |
| **Uv** | SRS | -0.203 | 0.042 | <0.001 (***) | -0.174 | 0.070 | <0.05 (*) | 0.030 | 0.081 | 127 | 0.37 | 0.7135 |

(e)

|  | **Var.** | **Stand. Regr-Coeff BL-CRF** | **SE** | **p-value (symbol)** | **Stand. Regr-Coeff End-CRF** | **SE** | **p-value (symbol)** | **Stand Regr-Coeff Difference** | **SE** | **df** | **t-value** | **p-value BL vs End** |
| --- | --- | --- | --- | --- | --- | --- | --- | --- | --- | --- | --- | --- |
| **Uv** | PF | -0.263 | 0.042 | <0.001 (***) | -0.196 | 0.035 | <0.001 (***) | 0.067 | 0.054 | 236 | 1.26 | 0.2106 |
| **Uv** | EF | -0.191 | 0.032 | <0.001 (***) | -0.171 | 0.029 | <0.001 (***) | 0.020 | 0.043 | 260 | 0.47 | 0.6420 |
| **Uv** | CF | -0.228 | 0.021 | <0.001 (***) | -0.188 | 0.021 | <0.001 (***) | 0.040 | 0.029 | 282 | 1.37 | 0.1733 |
| **Uv** | PSQI | -0.104 | 0.046 | <0.05 (*) | -0.162 | 0.034 | <0.001 (***) | -0.058 | 0.056 | 204 | -1.03 | 0.3020 |
| **Uv** | aR | -0.210 | 0.050 | <0.001 (***) | -0.292 | 0.037 | <0.001 (***) | -0.083 | 0.062 | 208 | -1.34 | 0.1813 |
| **Uv** | ICS | -0.380 | 0.048 | <0.001 (***) | -0.358 | 0.041 | <0.001 (***) | 0.022 | 0.063 | 237 | 0.34 | 0.7322 |
| **Uv** | SRS | -0.193 | 0.051 | <0.001 (***) | -0.219 | 0.038 | <0.001 (***) | -0.026 | 0.062 | 205 | -0.42 | 0.6765 |
| **Uv** | PF | -0.223 | 0.033 | <0.001 (***) | -0.180 | 0.032 | <0.001 (***) | 0.043 | 0.046 | 341 | 0.93 | 0.3518 |
| **Uv** | EF | -0.167 | 0.027 | <0.001 (***) | -0.171 | 0.027 | <0.001 (***) | -0.004 | 0.038 | 353 | -0.10 | 0.9171 |
| **Uv** | CF | -0.211 | 0.017 | <0.001 (***) | -0.176 | 0.020 | <0.001 (***) | 0.035 | 0.026 | 382 | 1.35 | 0.1784 |
| **Uv** | PSQI | -0.097 | 0.039 | <0.05 (*) | -0.155 | 0.032 | <0.001 (***) | -0.058 | 0.049 | 298 | -1.17 | 0.2421 |
| **Uv** | aR | -0.218 | 0.042 | <0.001 (***) | -0.298 | 0.035 | <0.001 (***) | -0.080 | 0.055 | 302 | -1.47 | 0.1436 |
| **Uv** | ICS | -0.376 | 0.040 | <0.001 (***) | -0.380 | 0.038 | <0.001 (***) | -0.004 | 0.054 | 338 | -0.08 | 0.9376 |
| **Uv** | SRS | -0.197 | 0.042 | <0.001 (***) | -0.246 | 0.035 | <0.001 (***) | -0.049 | 0.054 | 302 | -0.90 | 0.3681 |
